# A new long-proboscid genus of Pseudopolycentropodidae (Mecoptera) from the Middle Jurassic of China and its plant-host specializations

**DOI:** 10.3897/zookeys.130.1641

**Published:** 2011-09-24

**Authors:** ChungKun Shih, Xiaoguang Yang, Conrad C. Labandeira, Dong Ren

**Affiliations:** 1 College of Life Sciences, Capital Normal University, Beijing 100048, China; 2 Department of Paleobiology, Smithsonian Institution, National Museum of Natural History, Washington, DC 20013 USA; 3 Department of Entomology and BEES Program, University of Maryland, College Park, MD 20742 USA

**Keywords:** Pseudopolycentropodidae, fossil scorpionfly, new taxon, Jiulongshan Formation, proboscis, insect-plant associations, gymnosperms

## Abstract

We describe a new genus and species of Mecoptera with siphonate mouthparts, *Sinopolycentropus rasnitsyni* **gen. et sp. n.**, assigned to the family Pseudopolycentropodidae Handlirsch, 1925. The specimen was collected from late Middle Jurassic nonmarine strata of the Jiulongshan Formation in Inner Mongolia, northeastern China. The new material provides additional evidence for an early diversification of pseudopolycentropodids that was ongoing during the Middle Jurassic. This diversity also adds to the variety of known pseudopolycentropodids with tubular proboscides that apparently fed on ovulate fluids produced by Mesozoic gymnosperms.

## Introduction

The Pseudopolycentropodidae is an extinct and relatively nonspeciose family considered as phylogenetically basal to recent Mecoptera (Handlirsch 1925, Bode 1953, Willmann 1989, Carpenter 1992, Novokshonov 2002). Novokshonov (1997a) provided a general description of *Pseudopolycentropus latipennis* Martynov, 1927, then the best known species, and stated that all structural details of *Pseudopolycentropus latipennis* appear typical of the entire family except forewing shape. Grimaldi and colleagues (2005) comprehensively reviewed pseudopolycentropodid scorpionflies; they included additional details of seven previously described species of *Pseudopolycentropus* and their spatiotemporal distributions, and described the new taxa of *Pseudopolycentropus daohugouensis* Zhang, 2005, *Pseudopolycentropodes virginicus* Grimaldi & Fraser, 2005, *Parapolycentropus burmiticus* Grimaldi & Rasnitsyn, 2005, and *Pseudopolycentropus paraburmiticus* Grimaldi & Rasnitsyn, 2005. This contribution considerably expanded knowledge of the Pseudopolycentropodidae, and provided inferences on the possible diets of these bizarre, mid-Mesozoic insects.

Currently, the Pseudopolycentropodidae consists of thirteen described species assigned to three genera from the mid-Triassic to the mid-Cretaceous (Whalley 1985, Papier et al. 1996, Ansorge 1996, 2003, Novokshonov 1997a, 1997b, Grimaldi et al. 2005, Ren et al. 2009, Ren et al. 2010a). Five of these described species were based on fossils of forewings only, and one was based on fore- and hindwings. Of the remaining, seven species were described from wings and bodies; only five of which have preserved mouthparts, summarized in Table 1. They are *Pseudopolycentropus latipennis* (mid Late Jurassic of Karatau, Kazakhstan); *Pseudopolycentropus daohugouensis*, *Pseudopolycentropus janeannae* Ren, Shih & Labandeira, 2010, *Pseudopolycentropus novokshonovi* Ren, Shih & Labandeira, 2010 (all from the late Middle Jurassic of Daohugou, Inner Mongolia, China), and *Parapolycentropus burmiticus* (late Early Cretaceous of Kachin Province, northern Myanmar).

**Table 1.** Mouthpart, wing and antennal features of Mid-Mesozoic long-proboscid scorpionflies

Taxon	Localities and age	Body Length (mm)	Forewing		Proboscis Features					Clypeal area (mm2)	Antenna type	Sex
			Length (mm)	Width (mm)	Length (mm)	Width (mm)	Food tube dia. (mm)	Surface	Terminus			
Pseudopolycentropodidae												
*Sinopolycentropus rasnitsyni* gen. et sp. n.1	Daohugou, Inner Mongolia, China; Middle Jurassic (Bathonian–Callovian boundary)	5.5	6.1	2.4	1.9	0.1	0.027	Fine setae	Absent	??	Moniliform – compact with annulate hairs	♀
*Parapolycentropus burmiticus* Grimaldi & Rasnitsyn 20051	Tanai, Kachin, Myanmar; Early Cretaceous (Albian)	3.0	4.0	–	1.3	0.121	0.014	Setate, annulated	Lobate (Type 4)	—	Moniliform– aristate	♀
*Pseudopolycentropus latipennis* Martynov 1927	Aulie, Chimkent, Kazakhstan; Late Jurassic (Kimme-ridgian)	6.5	~9.8	~4.3	1.85	0.085	??	Fine setae	Tip broken	—	Filiform– compact	♀
*Pseudopolycentropus daohugouensis* Zhang 2005	Daohugou, Inner Mongolia, China; Middle Jurassic (Bathonian–Callovian boundary)	7.5	~7.0	~3.7	1.82	0.146	0.048	?	Tip broken	—	Moniliform	?
*Pseudopolycentropus janeannae* Ren, Shih & Labandeira 2010	Daohugou, Inner Mongolia, China; Middle Jurassic (Bathonian–Callovian boundary)	7.0 7.0	7.5 7.0	3.8 3.0	1.7 1.75	0.130 0.125	0.038 0.038	Fine setae	Absent	—	Filiform– compact	♀,♂
*Pseudopolycentropus novokshonovi* Ren, Shih & Labandeira 2010	Daohugou, Inner Mongolia, China; Middle Jurassic (Bathonian–Callo vian boundary)	7.0	8.0	3.9	1.5	0.13	0.038	Transversely ridged?	Tip broken	0.468	Filiform– compact	?
Mesopsychidae												
*Lichnomesopsyche gloriae* Ren, Labandeira & Shih 2010	Daohougou, Inner Mongolia, China; Middle Jurassic (Bathonian–Callo vian boundary)	28.0 23.0 23.0 –	25.0 24.0 27.0 24.0	7.0 8.0 8.0 8.0	9.9 8.9 8.9 10.1 >8.0 9.0	0.24 0.28 0.18 0.25 0.24 0.30	0.111 0.130 0.060 0.138 0.137 0.132	Coarse setae	Pseudo- labellum (Type 1)	0.387 (n = 7)	Filiform –broad	♀,♂
*Lichnomesopsyche daohugouensis* Ren, Labandeira & Shih 2010	Daohougou, Inner Mongolia, China; Middle Jurassic (Bathonian–Callo vian boundary)	>14	22.0	6.5	8.8	0.34	0.094	Coarse setae	Pseudo- labellum (Type 1)	0.361	Filiform broad	–
*Vitimopsyche kozlovi* Ren, Labandeira & Shih 2010	Pingquam, Hebei China, Early Creta- ceous (Barremian)	–	24.0	8.0	9.0	0.58	0.14	Smooth	Absent	0.436	–	–
Aneuretopsychidae												
*Jeholopsyche liaoningensis* Ren, Shih & Labandeira 2011	Huangbanjigou, Liaoning, China, Early Cretaceous (Barremian)	23.0	21.5	6.0	6.8	0.34	0.10	Smooth, annulated	V-shaped pseudolabellum (Type 2)	0.493	Filiform– compact	♂
*Aneuretopsyche minima* Rasnitsyn & Kozlov 1990	Aulie, Chimkent, Kazakhstan; Late Jurassic (Kimme-ridgian)	—	~10.5	—	4.7	0.18	0.060?	Fine setae, transversely ridged	Absent	—	Filiform– compact	?
*Aneuretopsyche rostrata* Rasnitsyn & Kozlov 1990	Aulie, Chimkent, Kazakhstan; Late Jurassic (Kimme-ridgian)	21.0	25.0	~7.1	7.3	0.21	0.075?	Fine setae, transversely ridged	Faint pseudo-labellum (Type 3)	–	Filiform–compact	♀
Nedubroviidae												
*Nedubrovia shcherbakovi* Bashkuev 2011	Isady, Vologda, Russia (Late Permian (Wuchiapingian)	~3.0	3.4	~1.3	>.035	NR2	NR2?	Fine setae,	??	–	??–	?

1 *Parapolycentropus* and *Sinopolycentropus* are the only two genera of Pseudopolycentropodidae known to have labial palps, albeit they are diminutive (Figs 2B, 2E, Grimaldi et al. 2005).

2 NR: not reported.

These five scorpionfly taxa are highly significant because *Pseudopolycentropus* possessed distinctive, elongate tubular, or siphonate, proboscides for surface fluid feeding on exposed plant fluids, such as the pollination drops of seed plants (Ren et al. 2009, Labandeira 2010). By contrast, one known species of *Parapolycentropus* bore a proboscis housing styliform structures. Although this genus, like other Pseudopolycentropodidae, probably imbibed ovulate fluids of seed plants (Grimaldi et al. 2005; Labandeira et al. 2007; Ren et al. 2009), the presence of one or perhaps two unserrated, styliform structures ensheathed within a proboscis is anomalous. One explanation is that this species of *Parapolycentropus* may have fed in a manner similar to a technical ink drawing pen with a central elongate wire for assisting laminar flow when food tube diameters are very narrow. Several genera of long-proboscid clades of Diptera have similar food tube arrangements, in which a thin, centrally positioned, rod-like but flexible hypopharynx extends to almost the proboscis terminus (Nagatomi and Soroida 1985, e.g. Figs 409, 425, 437). Such styliform processes lack serrations or apical piercing structures, and would be inconsistent with a blood feeding diet mentioned by Grimaldi et al. (2005).

Ren and colleagues (2009, 2010a, 2010b, 2011) reported that three families of mid Mesozoic, Eurasian Mecoptera had particularly elongate siphonate proboscides. They were the Mesopsychidae (*Lichnomesopsyche gloriae* Ren, Labandeira & Shih 2010, *Lichnomesopsyche daohugouensis* Ren, Labandeira & Shih 2010, and *Vitimopsyche kozlovi* Ren, Labandeira & Shih 2010), Aneuretopsychidae (*Jeholopsyche liaoningensis* Ren, Shih & Labandeira 2011), and the afore-mentioned Pseudopolycentropodidae (*Pseudopolycentropus janeannae* and *Pseudopolycentropus novokshonovi*). Structural details of these and other long-proboscid scorpionfly taxa are listed in Table 1, indicating together with other insect and botanical evidence, that these taxa fed on gymnospermous ovulate fluids such as pollination drops and likely engaged in pollination mutualisms with their host plants (Ren et al. 2009, Labandeira 2010).

The long proboscides of Mecoptera, in addition to other clades such as nemestrinid flies, seem to have originated during a 15 million-year interval during the mid Jurassic from a 170 to 155 Ma (Ren et al. 2009), indicated in part by earlier, Late Triassic pseudopolycentropodids lacking proboscides. However, this burst of long-proboscid origination is preceded by two considerably antecedent lineages that bear small but long proboscides, both from the Permian of Russia: the relatively small neuropteran Permithonidae, *Tschekardithonopsis*, from Early Permian Chekarda of Perm Province (Labandeira 2010), and the even smaller, mecopteran Nedubroviidae from Late Permian Isady of Vologda Province (Bashkuev 2011). Although much poorly known than the aneuretopsychine Mecoptera of the later mid Mesozoic, both the Late Permian and mid Mesozoic long-proboscid assemblages considerably antedate the similar and independent associations of nectar-feeding by flies, moths, and beetles on angiosperms several tens of millions of years later (Grimaldi 1999, Labandeira et al. 2007, Labandeira 2010). All three scorpionfly families became extinct during the later Early Cretaceous to mid-Cretaceous, coincident with global gymnosperm-to-angiosperm turnover (Ren et al. 2009, Labandeira 2010).

Adding to this inventory of long-proboscid scorpionflies, we recently collected a well-preserved fossil pseudopolycentropodid from the Middle Jurassic Jiulongshan Formation in Daohugou Village, Ningcheng County, Inner Mongolia, China. Based on its unique combination of antennae, mouthparts, and wing venation, a new genus and species is erected herein.

## Geological and paleobiological context

The Jiulongshan Formation is a lacustrine sequence that crops out near Daohugou Village, Shantou Township, Ningcheng County, in Inner Mongolia of northeastern China (41°19.532' N, 119°14.589' E) (Ren et al. 1995, Ren et al. 2002). The section at Daohugou is composed of grey tuffaceous sandstone and sandy mudstone. The paleoenvironment reconstructed for this locality was a volcanic region with montane streams and lakes (Ren et al. 2002). Daohugou has yielded an abundant and diverse insect fauna in addition to the Mecoptera, (Ren et al. 2010c) consisting of complete specimens of Ephemeroptera, Odonata, Plecoptera, Blattodea, Grylloblattida, Dermaptera, Orthoptera, Phasmatodea, Heteroptera, Sternorrhyncha, Neuroptera, Raphidioptera, Coleoptera, Hymenoptera, Diptera, Trichoptera and Lepidoptera. Apart from insects, Daohugou also has produced spiders, freshwater conchostracans, salamanders, feathered dinosaurs, pterosaurs, and mammals.

There is considerable evidence for a diverse local flora at Daohugou, which was important for plant interacting insects, such as pseudopolycentropodid scorpionflies. The Jiulongshan Formation has provided evidence for the floral composition of the surrounding forests. These forests were dominated by arborescent seed plants, principally Coniferopsida (*Pityophyllum*, *Rhipidiocladus*, *Elatocladus*, *Schizolepis*, *Podozamites*), Ginkgopsida (*Ginkgoites*, *Ginkgo*, *Baiera*, *Czekanowskia*, *Phoenicopsis*), Cycadopsida (*Pseudoctenis*, *Zamites*), and Bennettitopsida (*Anomozamites*) (Mi et al. 1996). Lower statured plants, including herbaceous ground cover, consisted of Lycopsida (*Lycopodites*, *Selaginellites*), Sphenopsida (*Equisetum*), and Filicopsida (*Todites*, *Coniopteris*) (Mi et al. 1996). These paleobotanical data were interpreted as indicating humid and warm-temperate climate (Tan and Ren 2002), although ecological amplitudes of specific habitats have yet to be determined. Verifiable 40Ar/39Ar and SHRIMP 238U/206Pb dating shows that the age of the volcanic rocks overlying the Daohugou fossil-bearing beds is ca. 164-165 Mya, and consequently the age of this fossil-bearing beds is slightly older than or equal to 165 Mya (Chen et al. 2004). Combined with the above-mentioned composition of insect fauna and conchostracans, the age of Daohugou biota is considered as Middle Jurassic (Zhang et al. 1987, Ren et al. 1995, 2002, Wang et al. 2000, Shen et al. 2003), equivalent to the Bathonian–Callovian boundary interval (Ogg et al. 2008).

## Materials and methods

**Figure 1.**
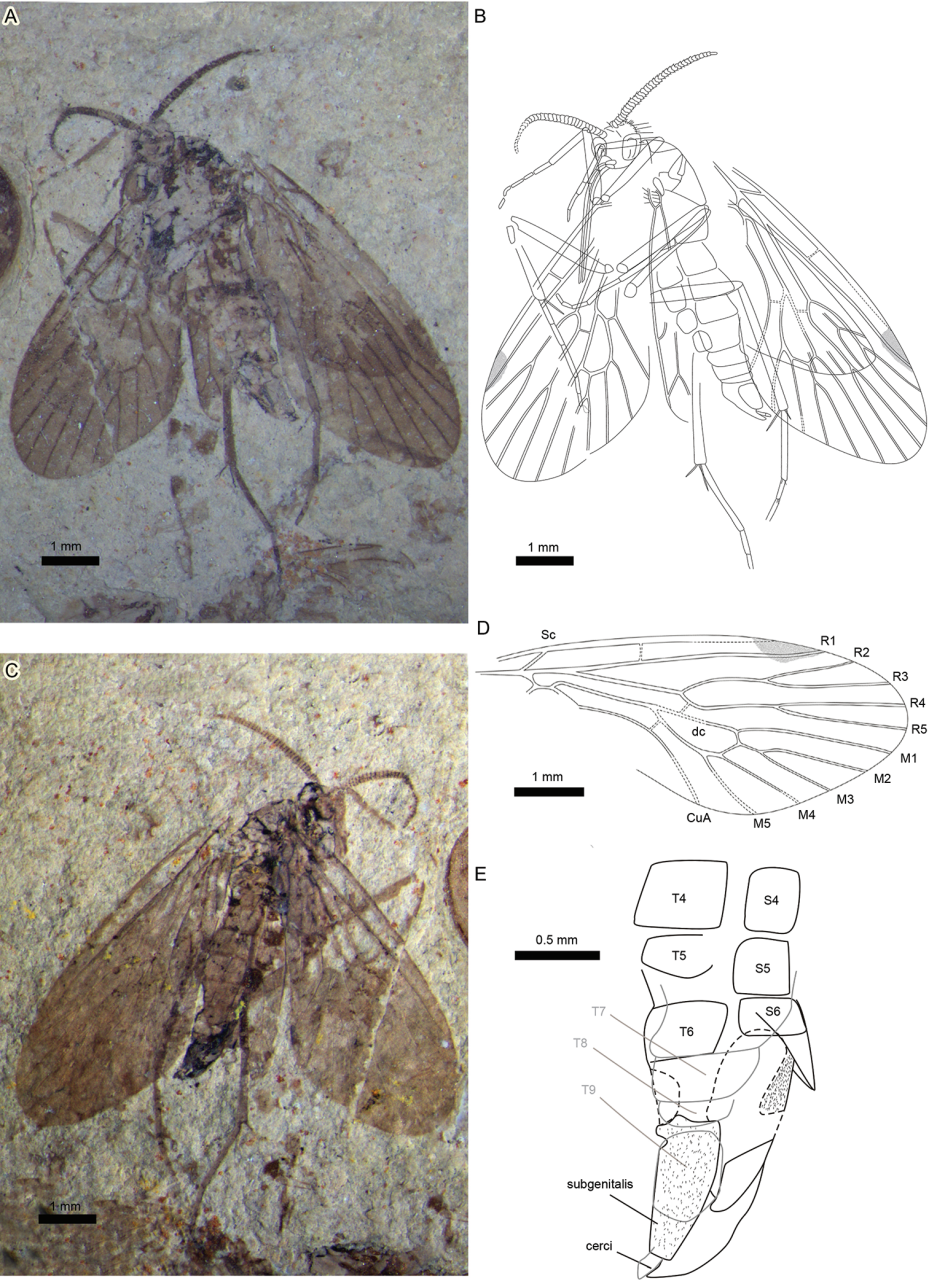
Photographs and line drawings of holotype *Sinopolycentropus rasnitsyni* gen. etsp. n. (specimen no. CNU-MEC- NN2010044 p/c) **A** Digital image of part, no.CNU-MEC-NN2010044p **B** Line drawing of part, no.CNU-MEC-NN2010044p **C** Digital image of counterpart, no.CNU-MEC-NN2010044c **D** Line drawing of forewing venation, no.CNU-MEC-NN2010044p **E** Line drawing of abdomen and terminalia, no.CNU-MEC-NN2010044c. Scale bars: 1.0 mm or 0.5 mm as shown in figures..

**Figure 2.**
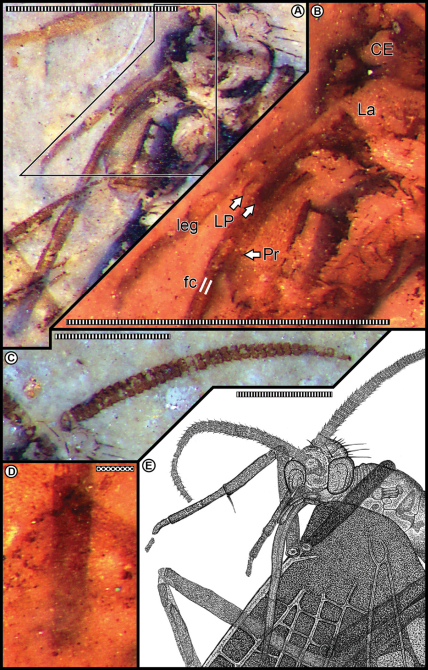
**A** Head, proboscis, and associated mouthparts **B** Mouthpart detail enlarged from template in **A**, showing base of proboscis (Pr), the tips of both labial palps (LP) at white arrows, labrum (La), and compound eye region (CE) **C** Right antenna **D** Proboscis tip, observed through the wing membrane **E** Camera lucida drawing of head, proboscis and associated mouthparts in **A** using a variety of light sources and angles. Scale bars: stippled, 0.1 mm; striped, 1.0 mm.

This study is based on a fossil specimen (CNU-MEC-NN-2010044 p/c), with part and counterpart, housed in the fossil insect collection of the Key Lab of Insect Evolution and Environmental Changes, College of Life Sciences, Capital Normal University, Beijing, China (CNUB; Dong Ren, Curator). The specimen was examined dry or under alcohol using a Leica M165 C dissecting microscope, and illustrated with the aid of a drawing tube attachment. Photographs of specimens were taken by Leica dfc500 and line drawings in Figure 1 were made by CorelDraw 12. The drawing in Figure 2 was done as a camera lucida tracing that subsequently was inked on polyester film and then reduced in size. Illumination for the drawing of this specimen consisted of three types of light and variation in light angle and origin for accentuation of morphological features, such as wing venation, that normally were difficult to observe and typically unavailable at lower magnification microscopes. The morphological terminology used herein is that of Grimaldi et al. (2005).

## Systematic paleontology

### Family Pseudopolycentropodidae Handlirsch, 1925

#### Genus Sinopolycentropus

Shih, Yang, Labandeira & Ren gen. n.

urn:lsid:zoobank.org:act:2C136D49-76A0-4BA4-8991-445366C376E5

http://species-id.net/wiki/Sinopolycentropus

##### Type species.

*Sinopolycentropus rasnitsyni* Shih, Yang, Labandeira & Ren, sp. n.

##### Etymology.

The generic name is a combination of the prefix “*Sino*” for China, and a shortened version, with the infix removed, of the type genus of its referred family, “*Pseudopolycentropus*”. The gender is masculine.

##### Diagnosis.

Forewing broad and rounded, triangular in overall shape, with base of Sc merging with R; R2+R3 forking earlier than R4+R5 forking. Antennae moniliform, compact, robust and thick, multiarticulate with annulate hairs. Distinct, multiarticulate labial palps and long occipital bristles also distinguish this taxon from all previously described Pseudopolycentropodidae except for *Parapolycentropus* Grimaldi & Rasnitsyn 2005.

##### Remarks.

This genus can be assigned to the Pseudopolycentropodidae by a short Sc, simple R1, Rs with four branches, M with five branches, a dc cell present, and a simple CuA. It can be differentiated readily from all other genera of Pseudopolycentropodidae by the base of Sc merging with R, R2+R3 forking earlier than R4+R5, and moniliform antennae consisting of compact, robust, relatively short, articles with annulate hairs. Distinctive labial palps and long occipital bristles also distinguish this taxon from all previously described Pseudopolycentropodidae except for *Parapolycentropus*. In addition, body length of *Sinopolycentropus* (5.5 mm) is shorter than that of *Pseudopolycentropus* (6.5 to 7.5 mm), but longer than that of *Parapolycentropus* (3.0 mm).

#### Sinopolycentropus rasnitsyni

Shih, Yang, Labandeira & Ren sp. n.

urn:lsid:zoobank.org:act:03B528B3-C5B9-45AB-9CF9-D046DD5028A6

http://species-id.net/wiki/Sinopolycentropus_rasnitsyni

Figs 1 ,2

##### Material.

Holotype, an almost complete specimen with well-preserved body and wings, female, part and counterpart, No.CNU-MEC-NN-2010044 p/c, is housed in the fossil insect collection of the Key Lab of Insect Evolution and Environmental Changes, College of Life Sciences, Capital Normal University, Beijing, China.

##### Etymology.

The specific name is dedicated to Dr. Alexandr Rasnitsyn for his contribution to paleoentomology and his recognition, with M. V. Kozlov, of the first fossil scorpionfly (*Aneuretopsyche rostrata*) with a documented long proboscis in 1990 (Rasnitsyn and Kozlov 1990).

##### Diagnosis.

As for the genus by monotypy.

##### Description.

A complete, small, female insect (Figs 1A-C); body length (excluding antennae and proboscis) 5.5 mm. Both forewings well-preserved, but hindwings only partially preserved, obscured due to overlap with forewings, thorax and abdomen.

Head and mouthparts. Head capsule spheroidal–prolate, prolonged anteriorly, housing prominent, hemispheroidal, and bulbous compound eyes (Figs 2A, E). Occipital region invested with conspicuously projecting, long bristles and smaller setae. Antennae 2.0 mm long, moniliform, compact and thick, with annulate hairs (Fig. 2C); about same length as proboscis. Each antenna consists of a basal scape and ca. 40 articles; each article bears hairs especially noticeable in profile along its distal annulus; proximal articles about twice as wide as long, distal articles equant. Undefined clypeal region evident below antennal base insertions and above the labrum (Fig. 2B). Mouthparts consistent with previously documented combination for pseudopolycentropodids (but see comments in discussion section below). There is the typical absence of mandibles and maxillary region, and presence of labral and labial elements; in part represented by discernable palps consisting of three articles (Figs 2B, E). Labrum triangular and inconspicuous. A long, decurved, siphonate proboscis 2.0 mm long, labially derived, occurring in an anatomically downturned position that lacks external cuticular ornamentation but bears very fine setae. Proboscis siphon diameter ca. 0.10 mm; housing an inner, eccentrically positioned food canal ca. 0.027 mm in diameter. Proboscis terminus lacks absorptive structures, such as pseudolabellae, related to feeding (Fig. 2D). Two, short labial palps present, adjacent and lateral to the proboscis base, each 0.5 mm long; about one-fourth proboscis length (Fig. 2B). Labial palps composed of three articles, the distal article slightly clavate, with a smooth, rounded terminus, the proximal articles thinner, the proximal-most attached to an enlarged labial area at the ventral base of the head capsule.

Thorax and legs. In lateral aspect pronotum short and neck-like; mesonotum broad, scutellum narrow, metanotum slightly shorter than scutum. Legs entirely covered with pubescence. Right foreleg originating from small, round coxa; long and slender femur (overlapping with thorax) and tibia (overlapping with head); left foreleg (overlapping with mouthparts) intersecting basitarsus of right foreleg and touching left antenna; tibia with at least two apical spurs. Midleg originating from small, round coxa; long and slender femur and tibia; tibia with at least 1 apical spur, tarsi of midleg 5-segmented, basitarsus longest, pretarsus with 1 evident claw. Hindleg originating from round coxa; long and slender femur and tibia; tibia with at least two, long, apical spurs. Tarsi of hindleg 5-segmented, basitarsus longest; length ratio of tibia and basitarsus 1:0.54 for left hind leg. Right hindleg disarticulated between femur and tibia.

Wings. Forewing broad, 6.1 mm long by 2.4 mm wide; length/width ratio 2.5; apical margin rounded (Figs 1A-D). (By comparison, the forewing length/width ratio is 2.0–2.3 for *Pseudopolycentropus janeannae* and 2.05 for *Pseudopolycentropus novokshonovi.*) Membrane covered in macrotrichia. Sc short, without anterior branches; base of Sc merging with R apex; Sc reaching C considerably before than Rs origin. Humeral vein absent. Crossvein c–r perpendicular to both R1 and C, just before wing midsection. R1 rectilinear at base, slightly arched toward C near wing midsection, coursing into the distinct pterostigma. Rs stem rectilinear. R2+R3 stem abruptly bent at crossvein r–m, then slightly arched toward C, with 2 long branches, R2 and R3. R2+R3 stem forking earlier than R4+R5; R4 longer than R2; R5 longer than R3. M forking slightly before that of Rs. Thyridium untraceable. M with 5 branches; M4+5 forking somewhat before the anterior M1+3 branch; M2+3 forking at about the same level as R4+R5 forking; M2+3 stem short and distinct. A crossvein between M4+5 stem and CuA, m–cua, near basal dc cell but present after M forking. M+CuA stem distinctly arched. M+CuA forking before R forking into R1 and Rs. Posterior wing margin almost rectilinear. Hindwing much smaller than forewing, but of similar shape. Right hindwing with only part of R2+R3 forking to R2 and R3; distal part of R4 and R5 preserved and left hindwing with a very short, terminal R1; basal Rs and part of R2+R3 forking to a preserved R2 and R3. Distal halves of fore- and hindwings suffused, pterostigma darkened (Figs 1A, 1B, 1D).

Abdomen. Abdomen elongate, tapering apically, with 9 visible segments. Basitergum (T1) fused to metathorax, segments 2-5 distinctly broad. Subgenitalis rectangular in shape and cerci visible (Figs 1B, 1C, 1E).

##### Locality and stratigraphic horizon.

Daohugou Village, Shantou Township, Ningcheng County, Inner Mongolia, China; Jiulongshan Formation, Middle Jurassic (Bathonian–Callovian boundary interval).

## Discussion

Three aspects of this discovery are significant for understanding the ecological roles of Pseudopolycentropodidae with plants in the local ecosystem at Daohugou. First is recognition of the distinct morphological features that separate *Sinopolycentropus* gen. n. from all other coexisting pseudopolycentropodid taxa. Second are the implications that the unique antennal and especially mouthpart modifications have for host-plant use. Last is the importance of rarity in understanding the pollinator associations in an increasingly well-documented, preangiospermous ecosystem from the Middle Jurassic.

### Distinctiveness of *Sinopolycentropus* from other Pseudopolycentropodidae

This new, long-proboscid species is distinct from all other members of Pseudopolycentropodidae by several differentiating features. These differences are the base of the Sc vein merging with R vein; the R2+R3 vein forking earlier than that of the R4+R5 vein; and relatively short, moniliform, and robust antennae bearing hairs on their annulae. To date, all described pseudopolycentropodids have their R4+R5 vein forking earlier than that of their R2+R3 vein, except for *Pseudopolycentropus triasicus* Papier, Nel & Grauvogel-Stamm, 1996, which has its R4+R5 vein forking at variable levels vs. its R2+R3 vein (Papier et al. 1996).

The presence of small, distinctive, three-segmented labial palps is only shared with *Parapolycentropus burmiticus* (Grimaldi et al. 2005). We reinterpret the proboscis sheath and palps of *Parapolycentropus burmiticus* (Grimaldi et al. 2005) as labial, rather than maxillary, in origin (Ren et al. 2009), a tendency in other Antliophora, such as the Diptera (Chaudonneret 1990). However, the proboscis stylets in *Parapolycentropus*, absent in *Sinopolycentropus*, are likely derivatives of maxillary laciniae or galeae.

Typically, pseudopolycentropodids have filiform or moniliform antennae with a relatively high ratio of antenna length to unappendiculate body length. For example, *Pseudopolycentropus daohugouensis* was reported to have an antenna/body length ratio somewhat greater than 0.37 (2.8/7.5); *Pseudopolycentropus janeannae* with greater ratios of 0.5 (3.5/7) and 0.57 (4/7); and *Pseudopolycentropus novokshonovi* with an intermediate ratio of 0.43 (3/7). However, *Parapolycentropus burmiticus* has a distinctively different type of antennae, characterized by a funnel-shaped scape, a scoop-shaped pedicel, the basal five flagellomeres tapered in size and the apical eleven segments significantly more diminutive, forming an arista (Grimaldi et al. 2005), all of which amount to an antenna/body length ratio of 0.4 (1.2/3). By contrast, *Sinopolycentropus rasnitsyni* gen. et sp. n. possesses compact, moniliform, and robust antennae, but with a relatively smaller antenna/body length ratio of 0.36. Last, the presence of distinctive, short, three-segmented labial palps, laterally placed at the proboscis base, is a condition not found in other, palpless pseudopolycentropodids, except for *Parapolycentropus* (Grimaldi et al. 2005, Ren et al. 2009).

### Significance of antennal and mouthpart modifications

Structural features of the antennae and mouthparts may be relevant for seeking conspecifics and host plants. The unique characteristics of antennal shape, structure and length in *Sinopolycentropus rasnitsyni* might be associated with the sensory detection of mates or food sources. The presence of prominent hairs encircling the annular area of each antennal article may be involved in detection of conspecific pheromonal cues or specific chemicals from particular plant hosts. Currently, we cannot draw any conclusions regarding these suggestions, pending scanning electron or high-resolution light microscopy of the antennal hair setal bases and other features. Of more importance are the structure, size, and shape of the proboscis, which indicate that it would have been used for access and imbibition of pollination drops or similar ovulate fluids from a variety of smaller gymnospermous fructifications (Ren et al. 2009, Labandeira 2010). The purpose of comparatively short labial palps, shared only with one other Mid-Cretaceous amber taxon with similar mouthparts and presumed feeding on ovular fluids, could have been for sensory detection of appropriate chemical cues from particular host plants. Such cues would have included fructification exudations or pollination-drop scents, perhaps similar to that of extant insects and their cycad and angiosperm hosts (Pellmyr and Thien 1986, Terry et al. 2004).

### The rarity of pseudopolycentropodid pollinators

In the extensive collection of more than 250,000 fossil insect specimens from Daohugou at Capital Normal University, we currently have collected seven specimens of *Pseudopolycentropus janeannae*, one specimen of *Pseudopolycentropus novokshonovi*, and a single specimen of *Sinopolycentropus rasnitsyni*. This suggests that these three species are extremely rare compared to other, co-occurring insect taxa. Significantly, approximately 20 specimens of small, nonglossate moths also have been found in this collection, of similar size and ecological relationships with plants as the pseudopolycentropodids. This comparison indicates that the ecologically equivalent pseudopolycentropodids are considerably rarer, and by inference, may have had more specialized associations with host plants than those of moths. These low abundances indicate that rarity, indeed, was an important feature of the Middle Jurassic Jiulongshan insect fauna and flora, contrary to the viewpoint that rarity was only a feature of angiosperm-dominated biotas from the mid Cretaceous and younger (Vermeij and Grosberg 2010). In addition to the Pseudopolycentropodidae, it also should be noted that two other mecopteran lineages with long-proboscid, siphonate mouthparts—the Mesopsychidae and Aneuretopsychidae—also had likely associations with gymnospermous seed plants, including pollination mutualisms (Ren et al. 2009, Labandeira 2010). The variability of proboscis lengths and widths, diameters of proboscis food canals, and other associated features of the mouthparts, antennae and wings of these three lineages, summarized in Table 1, indicate a similar variety of accessible plant hosts. Although the proboscis of *Sinopolycentropus rasnitsyni* is slightly longer than other pseudopolycentropodid species, nevertheless it is within the range of other confamilial taxa, indicating that the Pseudopolycentropodidae were rare, specialized associates of gymnospermous plants during the Middle Jurassic at Daohugou.

## Supplementary Material

XML Treatment for Sinopolycentropus

XML Treatment for Sinopolycentropus rasnitsyni
